# Factors related to sedentary behavior in older adult stroke patients in China: a study based on decision tree and logistic regression model

**DOI:** 10.3389/fpubh.2024.1457151

**Published:** 2024-12-10

**Authors:** Shuxian Liu, Juan Li, Xi Chen, Xiaowen Jiang, Rong Tang, Yumei Lv

**Affiliations:** ^1^Department of Nursing, Harbin Medical University, Harbin, China; ^2^Department of Nursing, Affiliated Hospital of Changchun University of Traditional Chinese Medicine, Changchun, China

**Keywords:** sedentary behavior, older adult patients, cerebral apoplexy, logistic regression model, decision tree, influencing factors

## Abstract

**Objective:**

This study investigates the factors influencing sedentary behavior in older adult Chinese stroke patients using decision trees and logistic regression models.

**Methods:**

Convenience sampling method was employed to enroll 346 respondents aged ≥60 years with stroke from the Department of Neurology of three tertiary-level A hospitals in Heilongjiang province, based on the inclusion criteria. The Sedentary Behavior Questionnaire for Older Adults, the International Physical Activity Questionnaire Short Form (IPAQ-S), the Pittsburgh Sleep Quality Index (PSQI), the Self-Rating Depression Scale (SDS), and the Social Support Scale (SSRS) were used to assess sedentary behavior, physical activity level, sleep quality, depressive symptoms, and social support, respectively. Decision tree and logistic regression models were employed to analyze the factors related to sedentary behavior in older adult stroke patients.

**Results:**

Of the 346 respondents, 233 (67.3%) had sedentary behavior. The logistic regression model showed that education level (OR = 2.843, 95%CI: 1.219–6.626), BMI (OR = 3.686, 95%CI: 1.838–7.393), longest consecutive sitting time (OR = 3.853, 95%CI: 1.867–7.953), and sleep quality (OR = 3.832, 95%CI: 1.716–8.557) were identified as risk factors for sedentary behavior in older adult stroke patients, while drink alcohol (OR = 0.386, 95%CI: 0.184–0.809) and physical activity level (OR = 0.064, 95%CI: 0.030–0.140) were identified as protective factors for sedentary behavior. Besides, the decision tree model showed that physical activity level, longest consecutive sitting time, sleep quality, BMI, depressive symptoms, and age were associated with sedentary behavior. The sensitivity and specificity of the logistic regression model were 69.9 and 93.1%, respectively, and the area under the receiver operating characteristic (ROC) curve was 0.900 (95% CI: 0.863–0.938). The sensitivity and specificity of the decision tree model were 66.4, and 93.1% respectively, and the area under the ROC curve was 0.860 (95% CI: 0.816–0.904).

**Conclusion:**

Our findings indicated that physical activity level, longest consecutive sitting time, sleep quality, and BMI were key factors associated with sedentary behavior. To achieve the purpose of improving rehabilitation effect and quality of life, this study combining decision trees with logistic regression models was of high value in studying factors influencing sedentary behavior in older adult stroke patients.

## 1 Introduction

Stroke has emerged as the second leading cause of death globally, following ischemic heart disease, and is associated with a high morbidity, mortality, and disability rate (1). Heilongjiang province, located in a high-latitude area with long and cold winters, has the highest stroke prevalence in China, In 2019, of 5.07% among individuals over 35 years old—5 ~ 6 times higher than in the southern regions. The global increase in the older adult population has resulted in a higher prevalence of stroke among older individuals, with approximately 38% of strokes occurring in people aged 70 years and above. In 2021, the prevalence of stroke among the older adult population (60 years and older) in China is approximately 3.5–5.5%, ranking first among all age groups (2). This poses a significant health challenge for the older adult population worldwide. The fatigue, weakness, and decreased physical functioning that often follow a stroke significantly reduce the activity level of stroke patients (3). Compared with younger and middle-aged stroke patients, older adult stroke patients are more likely to participate in sedentary activities because of the rapid decline in walking ability, increased complications, and a lack of social support associated with strokes. Sedentary behavior could potentially lead to localized circulatory disorders and increase the risk of thrombosis, thereby, it puts pressure on damaged blood vessels and the brain, worsening the symptoms of older adult stroke patients (4). Therefore, reducing sedentary behavior in older stroke patients presents an important, but significant, challenge for the Healthy China Initiative.

Sedentary behavior refers to the behavior of an individual who expends ≤1.5 metabolic equivalents (METs) of energy in a sitting, reclining, or lying position during all waking states (5). After stroke, patients may experience various complications that lead to a sudden reduction in physical activity and an increase in sedentary behavior (3), with total sedentary time increasing with age (6). Prolonged sedentary behavior results in decreased peripheral blood flow and elevated levels of triglycerides and high-density lipoprotein, increasing the risk of stroke recurrence and readmission (7), while decreasing the quality of patient life.

Sedentary behavior in stroke patients is influenced by a variety of factors, including general demographic factors such as age, education, and occupational status (8, 9), as well as disease, lifestyle, and mental health. Previous research has demonstrated that the presence of one or more chronic diseases, multiple comorbidities, and decreased walking ability in stroke patients is a significant contributing factor to elevated levels of sedentary behavior (10, 11). Furthermore, daily lifestyle behaviors such as smoking, alcohol consumption, and physical activity can also exert a substantial influence on sedentary behavior (12). Boyle et al. (13) found that poor sleep quality significantly heightened the risk of sedentary behavior, with a strong correlation between the two. The risk of sedentary behavior escalates with an increase in depressive symptoms and a decrease in social support (14, 15). The combined effect of these factors represents a significant threat to the overall quality of life of stroke patients. However, there have been no specific studies investigating sedentary behavior in older adult stroke patients, creating a gap in research. Therefore, it is crucial to examine the factors contributing to sedentary behavior in this population.

Logistic regression and decision models can be utilized to construct predictive models for influential factors (16, 17). Logistic regression focuses on the main effects of influential factors, but is limited in its ability to handle interactions. In contrast, decision tree modeling eliminates the covariance between variables and involves a series of logistic decisions, which deviates from traditional linear treatment. To identify the primary influencing factors, it is crucial to rank the importance of each indicator. However, the instability of classification effects, with the number of nodes and the lack of main effects analysis, severely restricts the interpretation of findings. Therefore, combining decision tree models and logistic regression models can offer complementary benefits and enhance analytical performance.

The use of logistic regression statistical analysis alone to investigate the factors influencing sedentary behavior is of relatively limited utility because of the inability to identify the most powerful factors. Therefore, this study utilized both logistic regression and decision tree models to explore the factors influencing sedentary behavior in older adult stroke patients, with the aim of establishing a foundation for developing personalized intervention programs to address sedentary behavior in older adult stroke patients in China.

## 2 Methods

### 2.1 Study design, participants, recruitment

A cross-sectional study was conducted from September 2023 to March 2024 in Heilongjiang, China. This study used a convenience sampling method to select three neurology departments from tertiary-level A hospitals in Heilongjiang Province, as the study sites. A previous study indicated that the sample size should be at least 5–10 times the number of variables (18). In this current study, there were a total of 26 observed variables. Accordingly, considering a potential 20% sample loss, the minimum required sample size would be 156. A total of 358 questionnaires were distributed during this study; 346 completed questionnaires with valid responses were returned, resulting in a recovery rate of 96.65% (346/358). Older adult stroke patients who met the inclusion criteria were chosen as the subjects. The inclusion criteria were defined as follows: (1) age ≥ 60 years, (2) met the Diagnostic Criteria for Various Major Cerebrovascular Diseases in China 2019 and have them confirmed by CT or MRI, (3) Functional Ambulation Category Scale (FAC) score of ≥2, (4) no signs of psychiatric disorders, normal hearing, clear cognitive abilities, and ability to communicate without any difficulties, (5) absence of conditions that would limit physical activity, such as severe myocardial infarction, myasthenia gravis, fractures, or other diseases, (6) no use of sleeping pills/sedatives and (7) provision of a signed informed consent form.

The Harbin Medical University Ethics Review Board approved the present subject recruitment and research at three neurology departments from tertiary-level A hospitals in Daqing (HMUDQ20231116221).

### 2.2 Method of data collection

#### 2.2.1 Measurement of sedentary behavior

The Sedentary Behavior Questionnaire for Older Adults was used to assess sedentary behavior and determine the longest consecutive sitting time duration. This questionnaire, developed by Ku et al. (19), aims to investigate the sedentary time of older adult stroke patients across 10 common areas of daily life over a period of 1 week, which is measured and analyzed via a question: “How much time have you spent on the following sitting or lying down activities in the past 7 days?.” If individuals engage in multiple activities simultaneously, one activity should be designated as the primary activity. For instance, if an individual watches TV and eats simultaneously, the two should be considered as a single activity, with participants choosing either ‘watching TV’ or ‘eating’. The 10 areas examined were as follows: watching TV shows or videos; using computers/internet devices (including tablets, mobile phones, and gaming consoles); reading books and newspapers; chatting or talking on the phone with friends or family members; taking public transportation; consuming meals (including three main meals, snacks, and tea/drinks); pursuing hobbies (such as listening to the radio, doing handicrafts, playing cards or chess); taking brief naps while seated; and being seated at work; and participating in other activities (e.g., attending church or temple). The average daily time spent on each category of sedentary behavior was calculated as (number of days per week) × (average time spent per day on those days)/7. The total sedentary time is the sum of the average daily time spent across all types of sedentary behaviors. The questionnaire demonstrated good reliability and validity, with test–retest reliability ranging from 0.740 to 0.920. This research refers to relevant literature both domestically and internationally (20, 21), which defines sedentary behavior as exceeding a total duration of 6 h per day.

#### 2.2.2 Measurement of physical activity

Physical activity levels were assessed using the International Physical Activity Questionnaire-short (IPAQ-S), which was developed by the International Physical Activity Measurement Working Group (22) in 2001 and translated into Chinese by Qu and Li (23). The reliability and validity of the Chinese version of the IPAQ were tested, yielding re-test coefficient and validity scale correlation coefficient values of 0.779 and 0.718 for physical activity, respectively. The metabolic equivalent of the questionnaire for walking was 3.3, for moderate-intensity activity it was 4.0, and for high-intensity activity, it was 8.0. The amount of physical activity was described using the metabolic equivalent (MET-min/w) according to the data processing method and grouping standard recommended by the IPAQ working group, and three levels of physical activity (walking, moderate, and vigorous) were calculated. The following formula was used: physical activity in 1 week = metabolic equivalent value of each type of physical activity × frequency per week (d/w) × time per day (min/d).

#### 2.2.3 Measurement of sleep quality

The Pittsburgh Sleep Quality Index (PSQI), revised by Liu et al. (24), was used to assess the sleep quality of older adult stroke patients during the previous month. Comprising 7 dimensions with a total of 19 items—including sleep quality, sleep onset latency, sleep duration, sleep efficiency, sleep disturbances, use of sedatives, and daytime dysfunction—each dimension is scored on a scale from 0 to 3 points, resulting in a total score ranging from 0 to 21 points. A total score below 11 indicates good sleep quality, while a score equal to or above 11 suggests poor sleep quality. The internal consistency reliability coefficient (Cronbach’s *α*) of this study was calculated to be as high as 0.838.

#### 2.2.4 Measurement of depression

The level of depression was assessed using the Self-Rating Depression Scale (SDS), which is characterized by its simplicity and straightforwardness, enabling it to effectively capture the subjective experience of depression in both outpatient and hospitalized patients. Wang and Chi (25) translated the scale into Chinese. The scale encompasses the following four dimensions: psychogenic affective symptoms, somatic disorders, psychomotor disorders, and depressive psychological disorders. It consists of a total of 20 items, with 10 items scored positively and the other 10 scored inversely. Each item is assigned a score ranging from 1 to 4 points, resulting in a total score range of 20 to 80 points. A total score between 53 and 62 indicates mild depression; scores ranging from 63 to 72 are classified as indicating moderate depression; while scores above 72 indicate severe depression, with higher scores indicating greater severity. In this study, a total score of ≥53 indicates the presence of depressive symptoms, while a score of <53 indicates the absence of depressive symptoms. The Cronbach’s *α* coefficient for the scale used in this study was found to be satisfactory at 0.900.

#### 2.2.5 Measurement of social support

The Social Support Rating Scale (SSRS), developed by Xiao (26), was used to assess the social support status of individuals in this study. This scale consists of the following three dimensions: subjective support, objective support, and support utilization, comprising a total of 10 items. Items 1–5 and 8–10 are assessed on a 4-point Likert scale ranging from 1 to 4, while items 6–7 are evaluated based on the “number of sources” scale, with one point awarded for each source. The cumulative score is calculated by summing the scores of individual items. A higher score indicates a greater level of social support. In our investigation, the internal consistency reliability coefficient (Cronbach’s *α*) for this scale was determined to be satisfactory with an estimated value of 0.833.

#### 2.2.6 Measurement of walking ability level

The functional ambulation category (FAC) scale was adopted to evaluate walking ability level (27). The FAC scale has six grades from 0 to 5, scored 0–5 respectively, where a higher score indicates better functional walking ability. Grade 0: unable to stand or walk; Grade 1: can walk within 10 m indoors with support; Grade 2: can walk 20 m indoors under supervision; Grade 3: can walk over 50 m independently indoors and ascend/descend 18-cm-high stairs twice independently; Grade 4: can walk over 100 m continuously, cross 20-cm-high obstacles and ascend/descend 10-floor stairs; Grade 5: can walk over 200 m continuously, ascend/descend stairs independently and at a speed over 20 m/min.

#### 2.2.7 Measurement of other variables

The additional variables encompass fundamental demographic and health-related data, were evaluated by using a general information questionnaire containing several questions, specifically age (<70 years and ≥70 years); gender (male and female); educational level (junior high school and below, junior high school and above); marital status (married, unmarried, divorced, and widowed); residence status (residing with children, relatives, caregivers, etc., or living alone); caregiver (yes or no); occupational status (retired and unemployed, farmers, and employed); monthly income (<3,000 or ≥3,000); BMI (healthy weight range: 18.5 ~ 23.9; overweight: ≥24; obese: ≥28); comorbid chronic diseases (0 ~ 2 or ≥3); family history of stroke (yes or no); limb function impairment (yes or no); smoking (yes or no); drinking alcohol (yes or no); history of falls (yes or no); and the number of complications (<1 or ≥1). Among them, the assessment criterion for alcohol consumption is to ask the patient, “In the past year, has the number of times of drinking alcohol per week been two or more?” If the answer is yes, it is judged as having drinking behavior; the complications encompass mild motor and balance disorders, memory impairment, dysphagia, as well as anxiety and depression.

### 2.3 Statistical analysis

First, we used frequency and percentage to illustrate the demographic characteristics of the participants and conducted a chi-square test for one-way analysis of variance to compare disparities between the different sedentary groups (yes or no).

Second, the significant variables identified in the univariate analysis were used to construct predictive models (logistic regression and decision tree) in order to determine factors influencing sedentary behavior among the older adult stroke patients. The decision tree model utilizes the CART algorithm, which effectively handles both discrete and continuous data. However, preprocessing is required to convert continuous variables into binary classification variables owing to the binary nature of decision trees. Based on previous literature of the same type, this study used the mean value as the threshold for division (17). The logistic regression model shows no multicollinearity, as indicated by the variance inflation factor (VIF) results, with none of the factors exceeding the critical value (Supplementary Table S1).

Finally, the predictive performance of the logistic regression and decision tree models was compared using the receiver operating characteristic (ROC) curve. The *Z*-value and corresponding *p*-value were calculated based on the area under the curve and standard deviation to assess whether a statistically significant difference existed between the two models. The significance level was set at *α* = 0.05.

All statistical analyses were conducted using SPSS 26.0 software. Two-tailed *p*-value of <0.05 was considered to indicate statistical significance.

## 3 Results

### 3.1 Characteristics of participants

The general demographic characteristics of the respondents are presented in Table 1. A total of 346 subjects were included in this study, out of which 233 subjects exhibited sedentary behavior. The one-way chi-square test revealed significant differences between the two groups in regard to age, educational level, marital status, residence status, caregiver, occupational status, BMI, comorbid chronic diseases, limb function impairment, smoking, alcohol consumption, complications, walking ability level, physical activity level, longest consecutive sitting time, depressive symptoms, social support, and sleep quality (*p*-value <0.05). Among the 233 subjects, 54.5% (127/233) were aged 70 years or older, 54.9% (128/233) were overweight or obese, 34.8% (81/233) had limb function impairment, 22.3% (52/233) smoked, 28.8% (67/233) consumed alcohol, and 46.8% (109/233) had at least one comorbidity.

**Table 1:** General characteristics of the respondents (*N* = 346).

Characteristics	Number of cases (*N* = 346)	Sedentary behavior (*N* = 233)	*X* ^2^	*P*-value
Age (in years)			10.117	0.001
<70	178(51.4)	106(59.6)		
≥70	168(48.6)	127(75.6)		
Gender			0.839	0.360
Male	193(55.8)	126(65.3)		
Female	153(44.2)	107(69.9)		
Education level			8.215	0.004
Junior high school and below	254(73.4)	160(63.0)		
Junior high school above	92(26.6)	73(79.3)		
Marital status			4.907	0.027
Married	249(72.0)	159(63.9)		
Unmarried/divorced/widowed	97(28.0)	74(76.3)		
Residence status			5.539	0.019
Living with others	289(83.5)	187(64.7)		
Living alone	57(16.5)	46(80.7)		
Caregiver			4.230	0.040
No	31(9.0)	26(83.9)		
Yes	315(91.0)	207(65.7)		
Occupational status			6.748	0.009
Retired and jobless	243(70.2)	174(71.6)		
Farmers and employed	103(29.8)	59(57.3)		
Monthly income			0.007	0.935
<3,000	59(17.1)	40(67.8)		
≥3,000	287(82.9)	193(67.2)		
BMI			18.868	<0.001
Normal	184(53.2)	105(57.1)		
Overweight and obesity	162(46.8)	128(79.0)		
Comorbid chronic diseases			9.750	0.002
0 ~ 2	295(85.3)	189(64.1)		
≥3	51(14.7)	44(86.3)		
Family history of stroke			0.030	0.863
No	244(70.5)	165(67.6)		
Yes	102(29.5)	68(66.7)		
Limb function impairment			13.216	<0.001
No	247(71.4)	152(61.5)		
Yes	99(28.6)	81(81.8)		
Smoking			4.329	0.037
No	257(74.3)	181(70.4)		
Yes	89(25.7)	52(58.4)		
Alcohol			4.943	0.026
No	233(67.3)	166(71.2)		
Yes	113(32.7)	67(59.3)		
History of falls (in 1 year)			0.179	0.672
No	330(95.4)	223(67.6)		
Yes	16(4.6)	10(62.5)		
Complications			9.717	0.002
<1	204(59.0)	124(60.8)		
≥1	142(41.0)	109(76.8)		
Walking ability level			4.325	0.038
2	39(11.3)	32(82.1)		
>2	307(88.7)	201(65.5)		
Physical activity level			94.547	<0.001
≤3000 MET-min/w	237(68.5)	199(84.0)		
>3000 MET-min/w	109(31.5)	34(31.2)		
The longest consecutive sitting time (hours)			71.905	<0.001
≤2	151(43.6)	65(43.0)		
>2	195(56.4)	168(86.2)		
Depressive symptoms			25.353	<0.001
No	227(65.6)	132(58.1)		
Yes	119(34.4)	101(84.9)		
SSRS (scores)			10.989	0.001
≤32	182(52.6)	137(75.3)		
>32	164(47.4)	96(58.5)		
PSQI			27.523	<0.001
Good	231(66.8)	134(58.0)		
Poor	115(33.2)	99(86.1)		

Exegesis: the social support scores were classified by mean value.

### 3.2 Results of logistic regression analysis

The variable assignments are shown in Table 2. The results of binary logistic regression analysis (Table 3) revealed that education level (OR = 2.843, 95%CI: 1.219–6.626), BMI (OR = 3.686, 95%CI: 1.838–7.393), drink alcohol (OR = 0.386, 95%CI: 0.184–0.809), physical activity level (OR = 0.064, 95%CI: 0.030–0.140), longest consecutive sitting time (OR = 3.853, 95%CI: 1.867–7.953), and sleep quality (OR = 3.832, 95%CI: 1.716–8.557) were significant factors influencing sedentary behavior among the older adult stroke patients (*p*-value <0.05). Furthermore, the model did not demonstrate a statistically significant association between depressive symptoms, social support, and sedentary behavior.

**Table 2:** Variable assignment table.

Variables	Assignment
Sedentary behavior	0 = No, 1 = Yes
Age	1 = <70, 2 = ≥70
Education level	1 = Junior high school and below, 2 = Junior high school above
Marital status	1 = Married, 2 = Unmarried/divorced/widowed
Residence status	1 = Living with others, 2 = Living alone
Caregiver	1 = Yes, 2 = No
Occupational status	1 = Occupational status, 2 = Farmers and employed
BMI	1 = Normal, 2 = Overweight and obesity
Comorbid chronic diseases	1 = 0 ~ 2, 2 = ≥3
Limb function impairment	0 = No, 1 = Yes
Smoking	0 = No, 1 = Yes
Alcohol	0 = No, 1 = Yes
Complications	1 = <1, 2 = ≥1
Walking ability level	1 = ≤2, 2 = >2
Physical activity level	1 = ≤3000 MET-min/w, 2 = >3000 MET-min/w
The longest consecutive sitting time (hours)	1 = ≤2, 2 = >2
Depressive symtoms	0 = No, 1 = Yes
SSRS (scores)	1 = ≤32, 2 = >32
PSQI	1 = Good, 2 = Poor

SSRS, social support; PSQI, Pittsburgh sleep quality index.

**Table 3:** Binary logistic regression analysis of factors influencing sedentary behavior in older adult patients with stroke.

Independent variables	SE	Wald *χ*^2^	*P*	OR	95%CI
Education level					
Junior high school above	0.432	5.854	0.016	2.843	1.219–6.626
Junior high school and below					1.000
BMI					
Overweight and obesity	0.355	13.502	0.000	3.686	1.838–7.393
Normal					1.000
Alcohol					
Yes	0.377	6.357	0.012	0.386	0.184–0.809
No					1.000
Physical activity level					
>3000 MET-min/w	0.396	48.125	0.000	0.064	0.030–0.140
≤3000 MET-min/w					1.000
The longest consecutive sitting time					
>2	0.370	13.311	0.000	3.853	1.867–7.953
≤2					1.000
PSQI					
Poor	0.410	10.747	0.001	3.832	1.716–8.557
Good					1.000

PSQI, Pittsburgh sleep quality index.

### 3.3 Results of classification and decision tree model

**Figure 1:**
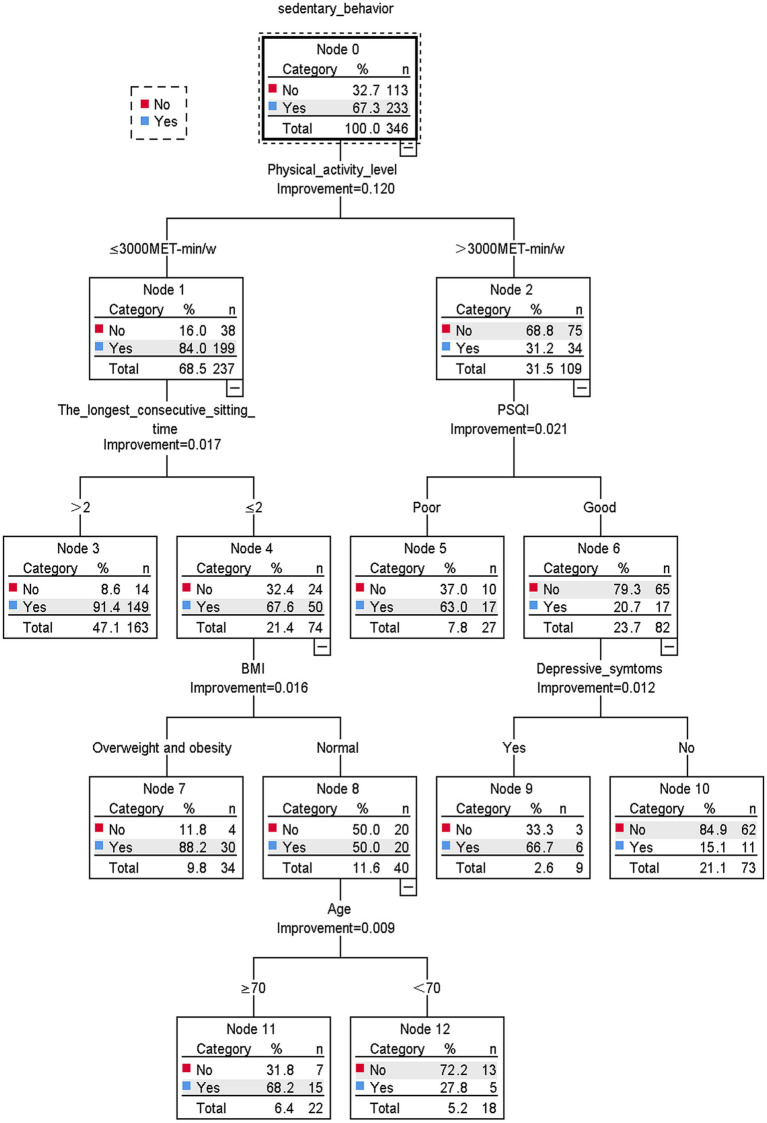
Classification and regression tree model (*N* = 346).

The results of the decision tree model are shown in Figure 1. Sedentary behavior was found to be mainly associated with physical activity level, longest consecutive sedentary time, PSQI, BMI, depressive symptoms, and age. The level of physical activity is the main determinant of sedentary behavior. Taking the physical activity level as the root node, the probability of sedentary behavior occurring in older adult stroke patients with a physical activity level of ≤3,000 MET-min/w is 84.0%. Older adult stroke patients with a physical activity level of ≤3,000 MET-min/w, longest continuous sedentary time of ≤2 h, normal BMI, and age ≥ 70 years old had a high probability of sedentary behavior (68.2%). In contrast, high levels of physical activity (>3,000 MET-min/w), good sleep quality, and no depressive symptoms were associated with a lower likelihood of sedentary behavior (15.1%).

### 3.4 Comparison of model prediction results

**Figure 2:**
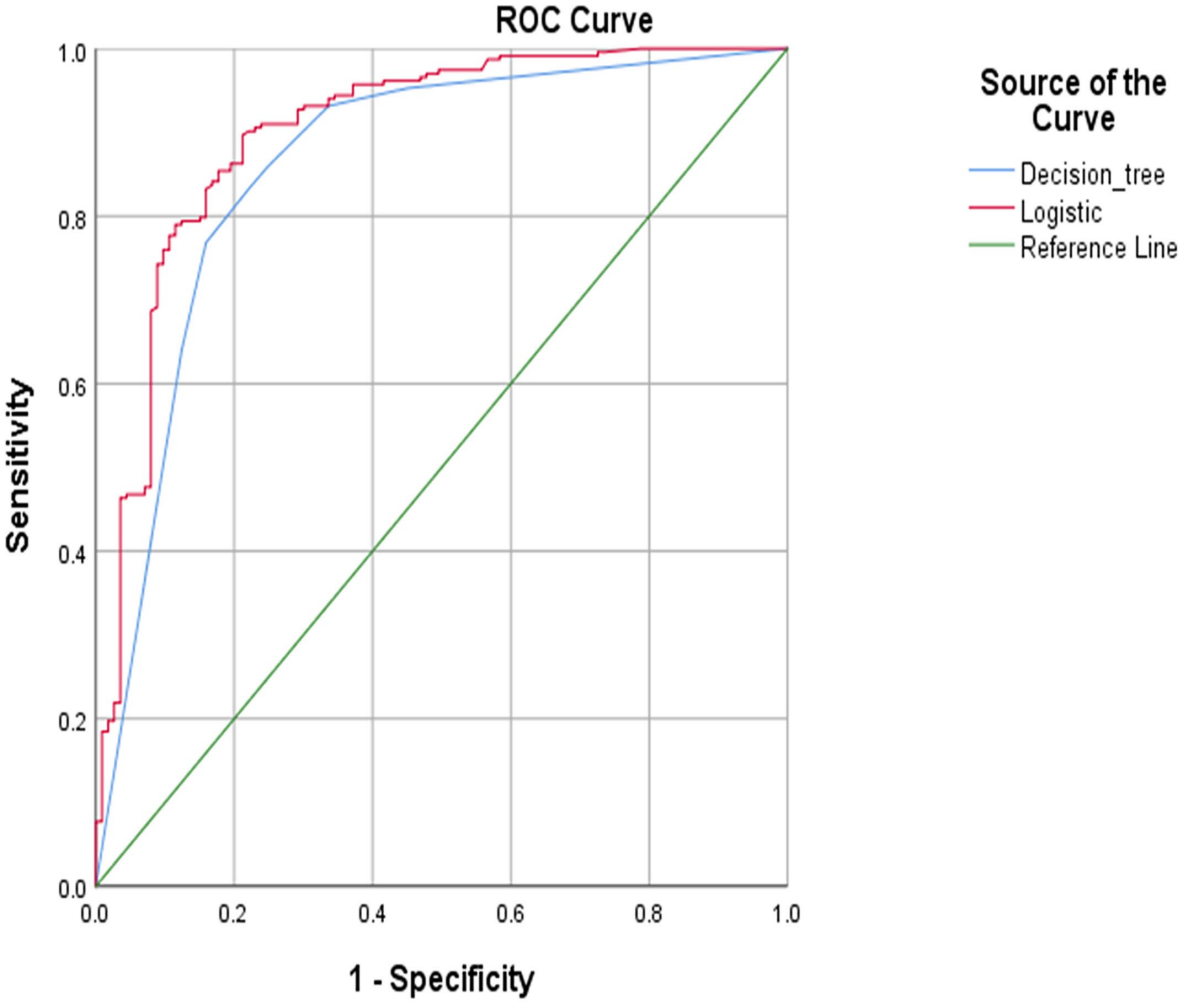
Receiver operating characteristic (ROC) curve predicted by decision tree and logistic regression model.

To compare the model’s predictive performance, separate ROC curves were plotted according to the prediction probabilities obtained by both models as test variables (Figure 2). The ROC curves for both models deviated significantly from the diagonal, indicating that the models possessed substantial predictive capability. The ROC curves of the two models demonstrate remarkable similarity, indicating comparable classification performance between the two models. However, the difference between the two models should be acknowledged. The decision tree model excluded the variables of education level and alcohol from the logistic regression model, while depressive symptoms and age were insignificant in the regression analysis.

The common influencing factors the logistic regression and decision tree models screened were physical activity level, the longest consecutive sitting time, sleep quality, and BMI. The logistic regression model achieved an area under the ROC curve (AUC) of 0.900 (95% CI: 0.863–0.938), while the decision tree model yielded an AUC of 0.860 (95% CI: 0.816–0.904). The logistic regression model exhibited a sensitivity of 69.9% and a specificity of 93.1%, while the decision tree model demonstrated a sensitivity of 66.4% and a specificity of 93.1% (Table 4). The two models exhibit a statistically significant difference (*Z* = 2.695, *p* = 0.007), and both demonstrated moderate predictive effects (0.8–1.0).

**Table 4:** Comparison of classification effectiveness of binary logistic regression model and classification decision tree model.

Model	AUC	Std. error	Asymptotic 95% Confidence Interval		Sensitivity (%)	Specificity (%)	Asymptotic Sig.
			Lower bound	Upper bound			
Logistic	0.900	0.019	0.863	0.938	69.9	93.1	<0.001
Decision tree	0.860	0.023	0.816	0.904	66.4	93.1	<0.001

## 4 Discussion

In this study, the prevalence of sedentary behavior among older adult stroke patients was found to be 67.3%. Lower than 79.6% of the research results of Fu et al. (28). The average self-reported total sedentary time was 6.96 ± 1.94 h/d, which was lower than the average total sedentary time measured by Duran et al. (29) using a hip-worn accelerometer 13.18 ± 1.34 h/d. This difference may be attributed to recall bias and reporting bias when measuring sedentary time through patient self-reporting. A Chinese study showed that the sedentary time of stroke patients in the community, as measured by questionnaire survey, was 7.10 ± 2.75 h/d (28), consistent with the findings of this study. One potential reason could be that stroke symptoms lead to decreased mobility and increased fatigue in patients, making it inconvenient for them to be mobile and therefore increasing their sedentary time. The study found that older adult stroke patients spend a lot of time being sedentary—mainly watching TV, using mobile phones, eating, and dozing. They spent less time reading books, newspapers, and magazines. Chinese author Shi Fangmin’s research also supports this finding (21). The reasons were considered to be that older adult stroke patients tend to also experience limb dysfunction, lack of peer support, a relatively narrow social circle, and generally low cultural level, resulting in less indoor and outdoor physical activity.

By combining logistic regression and decision tree models, we can investigate the variables that affect sedentary behavior. The important variables in the logistic regression model may differ from those in the decision tree model. For example, in the present study, according to the decision tree analysis, education level and alcohol consumption did not affect sedentary behavior. This may be because logistic regression models express correlations between variables, while decision tree models consider interactions and relationships between variables and detail the functional form of variables in each subclass, providing a wealth of information.

### 4.1 Influence of personal characteristics on sedentary behavior

In this study, gender, monthly income, and history of falls were not significantly associated with sedentary behavior. Older adult stroke patients with advanced age, higher levels of education, multiple comorbid chronic diseases, limb function impairments, and complications were found to exhibit elevated levels of sedentary behavior, which is consistent with previous studies (30, 31). As older adult stroke patients age, there is a gradual decline in their physical function and activity level, leading to an increase in sedentary behavior. Additionally, individuals with higher levels of education tend to engage more frequently in sedentary activities such as reading books and newspapers compared with those with lower educational attainment (21). Older stroke patients with multiple comorbid chronic diseases have significantly increased rates of sedentary behavior, suggesting that the presence of comorbid chronic diseases has a detrimental effect on sedentary behavior and, accordingly, that reducing the number of comorbidities may reduce the incidence of sedentary behavior. Good limb function is a critical prerequisite for engaging in physical activity. Impaired limb function can severely limit the ability of older stroke patients to exercise, leading to increased sedentary behavior (32). In addition, older stroke patients with more complications may have greater impairment in limb function and, consequently, are more likely to have longer periods of sedentary time.

The results of the univariate analysis in this study indicated no association between gender and sedentary behavior (*p* > 0.05), which was inconsistent with the findings of Li et al. (8). This discrepancy may be attributed to the fact that at this stage, Chinese stroke patients typically adhere to similar rehabilitation programs and medical advice throughout the recovery process, which contributes to the consistency of their daily activity patterns. Additionally, older adult stroke patients, regardless of gender, tend to engage in static activities such as watching TV and reading due to their physical limitations, which makes the gender difference in sedentary behavior less pronounced (33). The decision tree model demonstrated that age was a significant predictor of sedentary behavior in older adult stroke patients, a finding that aligns with the results of the study by Seo et al. (34). Although the univariate analysis yielded a statistically significant difference in sedentary behavior among older adult stroke patients of different ages (*F* = 10.117, *p* = 0.001), the final logistic model did not demonstrate a relationship. As they age, older adult stroke patients typically experience a decline in muscle strength, balance, and joint flexibility. This, coupled with their increased inclination toward traditional, static lifestyles and inadequate motivation for rehabilitation, contributes to the inclination toward sedentary behavior in older adult patients (35). Thus, when intervening in the sedentary behavior of older adult stroke patients, individualized rehabilitation programs must be developed according to the patient’s age, physical condition, and rehabilitation goals to enhance the patient’s mobility.

### 4.2 Physical activity level and sedentary behavior

We observed a correlation between the level of physical activity and sedentary behavior in older adult stroke patients, finding that those who regularly engaged in higher levels of physical activity had shorter sedentary times. This is consistent with the findings of Chan et al. (36), who reported that groups with higher total physical activity and moderate-to-vigorous physical activity exhibited lower levels of sedentary behavior. Raffin et al. (37) also found that individuals with higher levels of physical activity exhibited lower levels of sedentary behavior and suggested that a combination of the two could positively impact the negative correlation between grip strength and age. Additionally, research has indicated that replacing longer periods of sedentary behavior with more time spent in low-intensity physical activity and moderate-to-vigorous physical activity can increase the likelihood of healthy aging in older adults (38). One potential explanation for these findings is that sedentary behavior in older adult stroke patients may be influenced by impaired limb function, as well as the presence of fatigue and limitations in daily activities. Additionally, older adult stroke patients are more prone to recurrent episodes and hospital readmissions because of their advanced age, which may contribute to reduced physical activity levels because of their condition, thereby increasing sedentary behavior. Therefore, it is recommended that healthcare professionals involved in stroke care should focus on enhancing post-stroke rehabilitation and functional recovery training, refining stroke rehabilitation management, addressing the root causes of post-disease limb dysfunction, and promoting increased physical activity levels in order to mitigate the occurrence of sedentary behavior.

### 4.3 The longest consecutive sitting time and sedentary behavior

In short, individuals with a longer duration of uninterrupted sedentary time exhibited higher levels of sedentary behavior compared with those with a shorter duration. This is consistent with the findings of Guo and Wang (39), emphasizing the importance of interrupting the continuity of sedentary behavior as a crucial practical measure. The presence of comorbid chronic conditions, decreased levels of ambulatory capacity, and diminished social support are all potential contributors to an increase in maximum continuous sedentary time. The escalation of continuous sedentary time is associated with increased sedentary behavior, older adult stroke patients with a prolonged history of sedentary behavior are less inclined to engage in both indoor and outdoor physical activities, thereby perpetuating their sedentary lifestyle. Those with elevated levels of sedentary behavior may exhibit lethargy, fatigue, or impaired limb function, leading to decreased motivation for exercise and ultimately exacerbating their overall health status (40).

### 4.4 Sleep quality and sedentary behavior

Among older adult stroke patients, those with poor PSQI demonstrated elevated levels of sedentary behavior. You et al. (41) showed that sedentary behavior constitutes a risk factor for sleep disorders and that reducing sedentary time significantly enhances the quality of sleep in patients, which is consistent with the findings of the present study. Hofman’s et al. (42) research indicates that for every 30-min increase in sedentary behavior and a corresponding 30-min decrease in sleep time, there is an associated higher likelihood of poor perceived sleep quality (OR: 1.10 [1.04;1.16]) and daytime sleepiness (OR: 1.13 [1.00;1.28]). Post-stroke sleep disorders (SSD) are a common complication among stroke patients. Individuals who experience poor sleep quality exhibit a slower neurological recovery process and reduced motivation for physical activity, which leads to an increased prevalence of sedentary behavior (13). Furthermore, inadequate sleep quality leads to a greater propensity for patients to adopt negative coping mechanisms such as avoidance and non-cooperation during drug therapy, physical therapy, and rehabilitation training. This results in a delay in the rehabilitation process and impacts sedentary behavior levels (43). Therefore, medical personnel should pay due attention to addressing sleep problems among older adult stroke patients, enhance their understanding of their disease through psychological counseling, foster positive emotions, help create a quiet, comfortable and moderate light sleep environment for patients, encourage good sleep habits, improve their sleep quality, and reduce their sedentary time.

### 4.5 BMI and sedentary behavior

The study revealed that BMI is a significant risk factor for sedentary behavior. Webster-Dekker et al. (44) found a strong positive correlation between sedentary behavior and BMI (*p* < 0.001), indicating that higher BMI levels are associated with increased sedentary behavior. Our findings were consistent with those of this previous study. Hendrickx et al.’s (6) study revealed that individuals with obesity (mean BMI 30.1 kg/m^2^) exhibited a higher propensity for prolonged sedentary behavior in comparison with stroke patients with normal or overweight BMI (mean BMI 25.4–29.3 kg/m^2^). Individuals with a high BMI experience increased fatigue during physical activities, leading to more sedentary behavior. Prolonged sedentariness reduces the metabolic rate and energy expenditure. When energy intake exceeds expenditure, it contributes to further weight gain and an elevated BMI, perpetuating a cycle of adverse effects (45). In view of this, medical personnel should aim to impart high-quality health education to stroke patients to improve their levels of daily exercise, aiming to avoid prolonged sedentary activities and reduce the incidence of obesity by encouraging them to be more mobile and engage in regular physical activity.

### 4.6 Depressive symptoms and sedentary behavior

Older adult stroke patients comorbid with depressive symptoms exhibit prolonged sedentary behavior, consistent with the findings of Ashizawa et al. (46). Rehabilitation of stroke patients with limb dysfunction and speech disorders often involves the challenge of managing their heightened psychological stress and susceptibility to depression, which both significantly impact their overall quality of life. Studies have indicated that the prevalence of depression in stroke patients, attributed to the nature of their condition, ranges from 11 to 41% (47). Furthermore, elevated levels of depression can significantly impede the recovery process and prolong sedentary behavior (48). Depression, a key focus for psychological intervention, can be alleviated through interventions such as exercise therapy, pharmacotherapy, and cognitive behavioral therapy (49). Therefore, developing a comprehensive and personalized intervention strategy targeting depression to alleviate patients’ distress and stress, while maintaining their positive and optimistic mood, is an effective approach for promoting physical activity and reducing sedentary behaviors among older adult stroke patients (50).

### 4.7 Strengths and limitations

The study has several strengths. First, in the field of research on factors related to sedentary behavior of older adult stroke patients in China, this study fills the gap of existing studies to some extent, clarifies the influence of sleep quality, physical activity level and other factors on the sedentary behavior of older adult stroke patients, and provides new perspectives and data support for further in-depth understanding of the health behavior of this special population, which can help promote the progress of research in this field and provide important references and lessons for future related studies. Second, this study employed two statistical methods, namely, decision tree analysis and logistic regression modeling, to comprehensively identify influencing factors and determine the most influential combination of common factors.

One limitation of this study is that sedentary time was self-reported by patients, which may have introduced recall bias and affected measurement accuracy. Sedentary time was quantified in 15-min increments to mitigate this potential bias and corroborated through family member reports. Other random measurement errors are also limiting factors. These include the limitations of BMI (it does not measure fat distribution and muscle mass), the use of sleeping pills/tranquillizers and other medications, the possibility of specific comorbidities that may in themselves lead to an increase in sitting time (e.g., dizziness, balance disorders, locomotor problems beyond the stroke), and so on. Furthermore, due to the cross-sectional nature of this study, it was not possible to establish a causal relationship between influencing factors and sedentary behavior in the included older adult stroke patients. In addition, the sample size was relatively small. Future studies must utilize wider sample sources and conduct longitudinal research to elucidate the mechanisms underlying the development of sedentary behavior over time and establish causal relationships between influencing factors.

## 5 Conclusion

The results of this study show that the sedentary behavior of older adult stroke patients is influenced by a multitude of factors, including personal characteristics, disease features, and lifestyle. This study employed logistic regression and decision tree modeling to identify physical activity level, the longest consecutive sitting time, sleep quality, and BMI as the primary determinants of sedentary behavior in older adult stroke patients. These findings provide a foundation for future targeted interventions. In clinical practice, it is recommended that attention be directed toward high-risk groups as a priority to mitigate sedentary time and the prevalence of sedentary behaviors among stroke patients. It is recommended that medical and healthcare institutions strengthen community healthcare networks, provide comprehensive community healthcare services, and prioritize the rehabilitation of stroke patients. Older adult stroke patients are more susceptible to reduced physical activity because of the psychological impact and distress associated with their illness. Consequently, it is of paramount importance that medical professionals and family members provide psychological support to assist patients in developing a positive emotional mindset and engaging in health-promoting activities to mitigate the negative impact of sedentary behaviors.

## Data Availability

The original contributions presented in the study are included in the article/Supplementary material, further inquiries can be directed to the corresponding author.
