# Supplementary material for: Factors related to sedentary behavior in older adult stroke patients in China: a study based on decision tree and logistic regression model
Source: Front Public Health. 2024 Dec 10;12:1457151. doi: 10.3389/fpubh.2024.1457151 (PMC11666432; doi:10.3389/fpubh.2024.1457151)
Supplement: Supplementary file 1 [file Table_1.docx]

Supplementary Material

**Supplementary Table S1.** Diagnosis of covariance between variables

| Model | |  | N-St. Beta | Std. Error | St. Beta | t | P | Tolerances | VIF |
| --- | --- | --- | --- | --- | --- | --- | --- | --- | --- |
| 1 | (constant) | | 0.357 | 0.305 |  | 1.172 | 0.242 |  |  |
|  | Age | | 0.022 | 0.047 | 0.023 | 0.472 | 0.637 | 0.664 | 1.507 |
|  | Education level | | 0.119 | 0.048 | 0.112 | 2.493 | 0.013 | 0.804 | 1.243 |
|  | Marital status | | -0.059 | 0.054 | -0.056 | -1.084 | 0.279 | 0.604 | 1.656 |
|  | Living with family | | 0.142 | 0.063 | 0.112 | 2.263 | 0.024 | 0.662 | 1.511 |
|  | Care status | | 0.051 | 0.081 | 0.031 | 0.632 | 0.528 | 0.664 | 1.505 |
|  | Occupational status | | -0.020 | 0.052 | -0.020 | -0.391 | 0.696 | 0.630 | 1.588 |
|  | BMI | | 0.147 | 0.040 | 0.157 | 3.666 | 0.000 | 0.895 | 1.117 |
|  | Comorbid chronic diseases | | 0.027 | 0.058 | 0.020 | 0.466 | 0.641 | 0.861 | 1.162 |
|  | Physical dysfunction | | 0.106 | 0.072 | 0.102 | 1.471 | 0.142 | 0.341 | 2.929 |
|  | Smoking | | -0.049 | 0.049 | -0.046 | -1.013 | 0.312 | 0.791 | 1.264 |
|  | Alcohol | | -0.104 | 0.047 | -0.104 | -2.220 | 0.027 | 0.748 | 1.337 |
|  | Complications | | -0.085 | 0.061 | -0.089 | -1.400 | 0.163 | 0.403 | 2.480 |
|  | Walking ability level | | 0.097 | 0.074 | 0.066 | 1.314 | 0.190 | 0.655 | 1.527 |
|  | Physical activity level | | -0.383 | 0.049 | -0.380 | -7.871 | 0.000 | 0.701 | 1.426 |
|  | Maximum continuous sitting time for a single session | | 0.175 | 0.047 | 0.185 | 3.705 | 0.000 | 0.653 | 1.530 |
|  | Depressive symtoms | | -0.051 | 0.055 | -0.052 | -0.935 | 0.350 | 0.526 | 1.901 |
|  | SSRS | | 0.025 | 0.046 | 0.026 | 0.538 | 0.591 | 0.674 | 1.483 |
|  | PSQI | | 0.142 | 0.043 | 0.148 | 3.304 | 0.001 | 0.819 | 1.222 |
| a: implicit variable：sedentary behavior | | | | | | | | | |
